# Role of Cone-Beam Computed Tomography in the Management of Periodontal Disease

**DOI:** 10.3390/dj7020057

**Published:** 2019-06-01

**Authors:** V. Thomas Eshraghi, Kyle A. Malloy, Mehrnaz Tahmasbi

**Affiliations:** 1 Private Practice, Tualatin, OR 97062, USA; 2 Private Practice, Beaverton, OR 97006, USA; 3 Department of Periodontology, Oregon Health & Science University, Portland, OR 97201, USA; 4 Private Practice, Portland, OR 97266, USA; kmalloy@willamettedental.com; 5 Department of Diagnostic Sciences, Texas A&M College of Dentistry, Dallas, TX 75246, USA; arashlow@tamhsc.edu

**Keywords:** cone beam computed tomography (CBCT), 3D radiography, periodontal defects, periodontal diagnosis, furcation, intrabony defects

## Abstract

The goal of this paper was to review the current literature surrounding the use of cone beam computed tomography (CBCT) related to the diagnosis, prognostic determination, and treatment of periodontal diseases. A literature review was completed to identify peer-reviewed articles related to CBCT and periodontics. The results were filtered to pool only articles specific to CBCT and periodontal diagnosis, prognosis, and treatment/outcomes. The articles were reviewed and findings summarized. Author’s commentary on technological advances and additional potential uses of CBCT in the field of periodontics were included. There is evidence to suggest that CBCT imaging can be more accurate in diagnosing specific periodontal defects (intrabony and furcation defects), and therefore be helpful in the prognostic determination and treatment planning. However, at this time, CBCT cannot be recommended as the standard of care. It is up to the individual clinician to use one’s own judgment as to when the additional information provided by CBCT may be beneficial, while applying the As Low As Reasonably Achievable (ALARA) principle. With continued technological advances in CBCT imaging (higher resolution, reduced imaging artifacts, lower exposure, etc.) the author’s believe that CBCT usage will become more prominent in diagnosis and treatment of periodontal diseases.

## 1. Introduction

Periodontal diseases have high prevalence in both developed and developing nations affecting upwards of 20 to 50% of global populations [1]. Established risk factors associated with periodontal disease include diabetes, smoking, genetic pre-disposition, stress, medications, and other factors. Recent evidence has shown strong association of periodontal disease with cardiovascular disease, diabetes, and adverse pregnancy outcomes [2].

Poorly controlled periodontal diseases are characterized by attachment loss, bone loss and in the most severe progression, tooth loss. The first step in disease management is proper diagnosis. Diagnosing periodontal defects classically has relied upon interpretation of two-dimensional (2D) radiographs combined with a clinical evaluation consisting of probing depths, bone sounding and tracking indices related to marginal inflammation, bleeding on probing and purulent discharge [3,4,5,6]. 2D imaging modalities are easily acquired, high resolution and cost effective with minimal radiation exposure. However, they are not without limitations [7] and studies have indicated that intra-oral radiography underestimates bone loss due to projection and or observer errors [8,9,10]. While reliable, defects in the straight buccal, lingual, as well as furcations of affected teeth can be difficult or impossible to properly diagnose requiring further surgical extension for diagnosis and treatment [11]. While direct visualization via flap reflection is a viable treatment, it may result in an unproductive surgical outcome, extra clinical time, and an untimely discussion of alternative treatment options during surgery.

Cone beam computed tomography (CBCT) imaging is a powerful tool allowing for the diagnosis of three-dimensional (3D) structures and is well documented as a tool for accurate quantification and locating anatomic structures. With widespread use of CBCT technology and the ability to see circumferential regions of the mouth, it has been a natural hope that bony defects and furcations could become identifiable with less invasiveness and clearly visible as a patient education tool as well.

The aim of this paper was two-fold: (1) to review current evidence-based literature and determine the efficacy of CBCT for management of periodontal disease and (2) to discuss current limitations and possible future applications.

### 1.1. Current Evidence of CBCT and Periodontal Therapies

With the prevalence of CBCT, new research is emerging with evidence to support its role in periodontal treatment. Although the data is limited, what is available highlights the potential for a greater role of CBCT in periodontal therapy. The areas with the current best evidence to support the use of CBCT in periodontal therapy are advanced radiographic diagnosis and post-surgical evaluation.

The gold standard for periodontal examination has been, and continues to be, completion of a clinical evaluation (including probing depths, bleeding/suppuration on probing, mobility, attachment level, furcation involvement, etc.) and radiographic evaluation (bite wing and periapical images). This conventional assessment has long been the most accurate for diagnosis of periodontal disease, including the presence of intrabony defects and furcation involvement. Although the presence of intrabony defects and furcation involvement can be well identified with clinical evaluation and a standard 2D intraoral radiographic exam (IOR), evidence is emerging that CBCT imaging is a beneficial addition during the diagnostic process.

Braun et al. [12] have reported that CBCT is superior to IOR in the detection of intrabony defects and furcation involvement. Overall, correct identification of intrabony defects occurred 82.7% using IOR and 99.7% with CBCT. CBCT was also better at identifying furcation involvement (94.8%) compared to IOR (75.6%). Their conclusion was the addition of the third dimension significantly increased the accuracy of diagnosis with the CBCT. Brags et al. [13] had similar findings regarding the detection of dehiscence (46.8% versus 78.2%) and fenestration (25.7% versus 89.1%) when comparing IOR versus CBCT. Several other studies have concluded similar findings that CBCT is more accurate in the detection of intrabony defects and furcation involvement, that IOR also tends to underestimate the severity of each, and that there is greater inter-examiner agreement with CBCT when compared to IOR [14,15,16].

Walter et al. [17] studied the accuracy of conventional assessment, clinical exam with IORs, to conventional assessment with additional CBCT evaluation for determination of degree of furcation involvement and appropriateness of treatment planning of maxillary molars with furcation involvement. Maxillary molars were assessed following completion of initial non-surgical therapy. This included standard periodontal data collection (probing depths, attachment levels, mobility, etc.) with furcation involvement classified according to Hamp’s classification based off detection with a curved Nabers probe. The clinical evaluation was aided by IORs and a treatment plan was determined. Next, CBCT evaluations were completed, analyzed to determine degree of furcation involvement, and compared to the findings from the conventional assessment.

They found that the degree of furcation involvement determined by conventional assessment was accurate only 27% of the time, and this was mostly associated with degree III furcations. Conventional assessment overestimated the degree of involvement 29% of the time and underestimated it 44% of the time. Degree I furcations were most commonly overestimated, while degree II furcations were underestimated. They also found that the recommended treatment plan based off the conventional assessment was in agreement with the recommendation based off of the CBCT data on 41% of the time. For the remaining cases, the CBCT based treatment plans were more invasive 41% of the time and 18% less invasive than the treatment plans established by the conventional assessment. They concluded that the addition of CBCT data was beneficial for the assessment of furcation involvement and determination of a more appropriate treatment plan.

Additional studies have concluded that CBCT evaluation of furcation involvement, horizontal bone loss, and intrabony periodontal defects cannot only be properly identified but they can also be accurately measured. Walter et al. [18] found that maxillary molar furcation involvement was accurately assessed via CBCT evaluation when compared to intra-surgical findings 84% of the time, while underestimating 14.7% and only over estimating 1.3%. Padmanabhan et al. [19] determined that there was no statistical significance between CBCT and direct intra-surgical measurements regarding furcation height, width, and depth. Banodkar et al. [20] found that CBCT was highly accurate at both detection of periodontal defects and determination of type of defect while also very precisely being able to measure the vertical depths of the defects. Another study by Feijo et al. [21] reported that there was no statistical difference between measurements of horizontal bone loss when measured by either CBCT or direct intra-surgical measurements.

Another beneficial application of CBCT reported by Zhao et al. [22] was the ability to assess root concavities of first premolars and associated pattern of bone loss. They identified five types of roots concavities based on origination of the concavity. Type I had no concavity, Type II the concavity originated in the enamel, Type II was coincidence with the CEJ, Type IV below the CEJ (but in the top 2/3 s of the root), and Type V was within the bottom 1/3 of the root. The associated pattern of bone loss was classified as a Ramp, Plane, or Crater. Maxillary first premolars had a mesial concavity present 100% of the time with Type II most common at 35.7%. A distal concavity was only on 39.3% of the sites, with Type IV as the most common (14.2%). Mandibular first premolars had a much lower presence of concavities with only 42.5% noted on the mesial, which were evenly distributed between Type II-V, and 31.3% on the distal with Type IV most common (15%). The distribution of patterns of bone loss was interesting. They were consistent between the mesial and distal sites when divided by presence or absence of a concavity. A Ramp, Plane, and Crater was noted 3.17%, 30.6%, and 37.8%, respectively, at the mesial sites with a concavity present and 58.7%, 27.2%, and 14.1%, respectively, for mesial sites without a concavity. The distal sites had a very similar distribution with 31.9%, 27.7%, and 40.4%, respectively at sites with a concavity and sites without a concavity had 57.9%, 28.1%, and 14.0%, respectively.

Information this detailed is not always known, or at least not certain, following conventional assessment. The greater the detail of the information available, the better the prognostic assessment and treatment plan can be. The conclusion of a systematic review by Nikolic-Jakoba et al. [23] on CBCT for detection of intrabony and furcation defects was that insufficient evidence was available to support its use. While this conclusion may seem discouraging, it was mainly due to the fact that there is currently limited scientific literature at this time. They also noted that in certain cases it still might be clinically beneficial to implement CBCT when conventional assessment may be lacking.

Although the data is rather limited, it is beginning to highlight some specific clinical situations where CBCT may be a beneficial adjunct to conventional assessment. In addition to the previously discussed benefits, CBCT can aid treatment/surgical planning in several ways. CBCT can allow a clinician to locate and map vital structures, such as the inferior alveolar, lingual, mental, or greater palatine nerves [24,25,26], when planning surgical therapies. CBCT can be utilized for evaluation of biotype by measuring hard and soft tissue thickness of the alveolar process. It can also be used to detect facial plate thickness and for identification of a dehiscence or fenestrations over root surfaces. This information can aid in diagnosing altered passive eruption (based on crestal bone level in relationship with the CEJ of the teeth) and surgical planning, especially for advanced soft and/or hard tissue augmentation [27,28,29,30,31,32,33,34,35]. However, one of the most useful applications of CBCT assessment is for post treatment evaluation.

Traditionally, post-surgical evaluation would require surgical reentry to verify outcomes. This is not only invasive for the patient, but also time consuming, and therefore financially costly for the practitioner. In private practice, these surgical outcomes are typically assessed using the same parameters as the clinical evaluation (including probing depths, bleeding/suppuration on probing, mobility, attachment level, furcation involvement, etc.) and radiographic evaluation. However, CBCT evaluation of post-surgical treatment is rapidly moving to becoming the standard of care in both private practice and for reporting in periodontal literature [36,37,38,39]. In addition to the accuracy of CBCT imaging to detect and measure intrabony defect and furcation involvement as previously described, CBCT can be utilized to accurately assess bone levels via circumferential quantification and a traditional six-site method [40,41]. Notably, CBCT evaluation following regenerative surgical treatment of furcation involvement was recommended by the American Academy of Periodontology (AAP) Regeneration Workshop in 2015 [42].

Recently, the AAP released a Best Evidence Consensus (BEC) series of papers related to the use and application of CBCT in the field of periodontics. A panel of experts related to the use of CBCT and who had significant experience implementing CBCT in practice were brought together to review the available literature and provide a consensus statement regarding the uses of CBCT [43]. The conclusion regarding the use of CBCT in the diagnosis, treatment planning, and management of periodontal disease was that limited evidence is available to support its use as standard of care [44,45]. Conventional assessment was still considered the gold standard but that in certain situation (advanced intrabony and furcation defects, suspected endo-perio lesions, root resorption, etc.) CBCT imaging may be beneficial. It is up to the clinician to determine when CBCT imaging is of benefit and applying the “As Low As Reasonably Achievable” (ALARA) principle. Even though CBCT exposures are considered to be low exposure, if the information from an additional CBCT is not beneficial from a diagnostic, prognostic, or treatment management perspective, it would not follow the ALARA principle.

### 1.2. Technological Advances and Potential Uses of CBCT and Periodontal Therapies

As with all technology, new generations of dental in-office scanners aim improve upon legacy devices. The primary goals are to reduce radiation artifact, improve quantification of bone density, reduce radiation exposure and reduce equipment costs. As of the writing of this paper, new systems have arisen that allow modular upgradeability from 4 × 4 cm to 16 × 17 cm FOV. This allows clinicians to start with focused field of views keeping costs down and upgrading as necessary. Furthermore, voxel sizes have decreased as low as 75 μm with dual jaw capabilities up to 10 × 10 cm allowing for ultra-high resolution on a single scan versus multiple regional exposures.

New scanners employ elevated 120 KV power to increase image quality at similar doses, net reduction in energy and beam hardening artifacts and reducing overall radiation effective dose. Furthermore, the scanners offer scout view capabilities to reduce errant exposures and low dosage modes reducing effective dose with voxel sizes of 300 to 400 μm.

**Figure 1:**
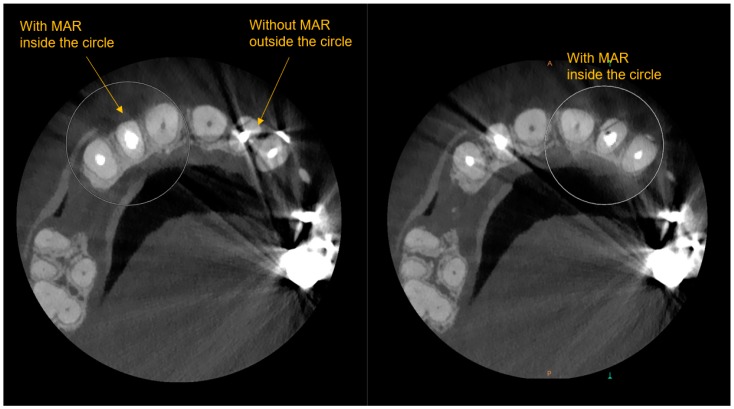
Axial views of CBCT with and without Metal artifact reduction (MAR) - (Carestream CS9600 MAR).

**Figure 2:**
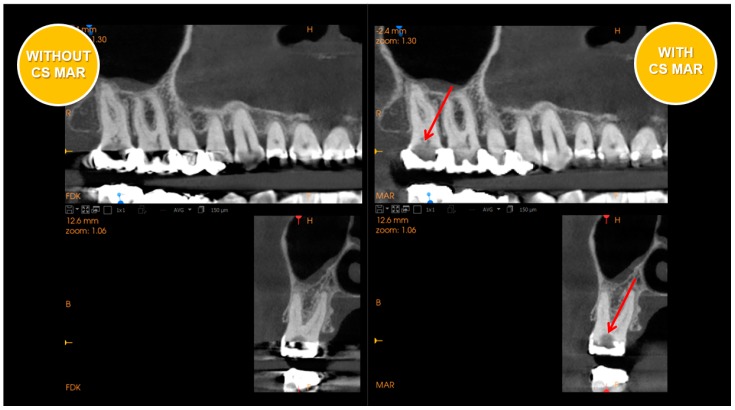
Sagital views of CBCT with and without Metal artifact reduction (MAR) - (Carestream CS9600 MAR).

Metal artifact reduction (MAR) employed in the latest generation of scanners is a very significant advancement. See Figure 1 and Figure 2 which have side by side comparisons of the improvements a metal artifact reduction filter offers. This potentially helps to confirm diagnoses in complex restorative cases and reduces the risk of misinterpretation.

The primary benefit will be in visualization of fractures, which greatly affect the periodontium and preclude regeneration of intrabony defects. Fractures generally occur in complex restored teeth and often are endodontically and periodontally involved leading to misdiagnosis without a metal scatter reduction filter.

**Figure 3:**
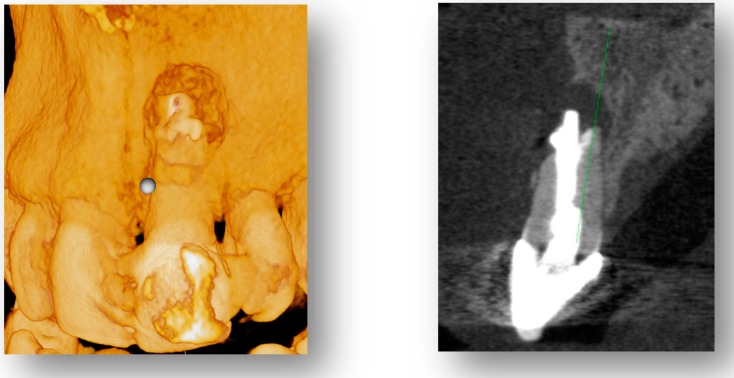
Three-dimensional (3D) rendering (**left image**) and CBCT cross-section (**right image**) a0 of periodontal defect with reduced scatter.

Reduced voxel size and MAR will enable improved 3D visualization of periodontal defects. This will aid tremendously in flap design to understand severity and extension of the lesion and to maintain blood supply to thin segments of remaining bone as visualized at the coronal aspect of the cross-section slide in Figure 3. This is also a powerful tool in visual patient treatment planning.

**Figure 4:**
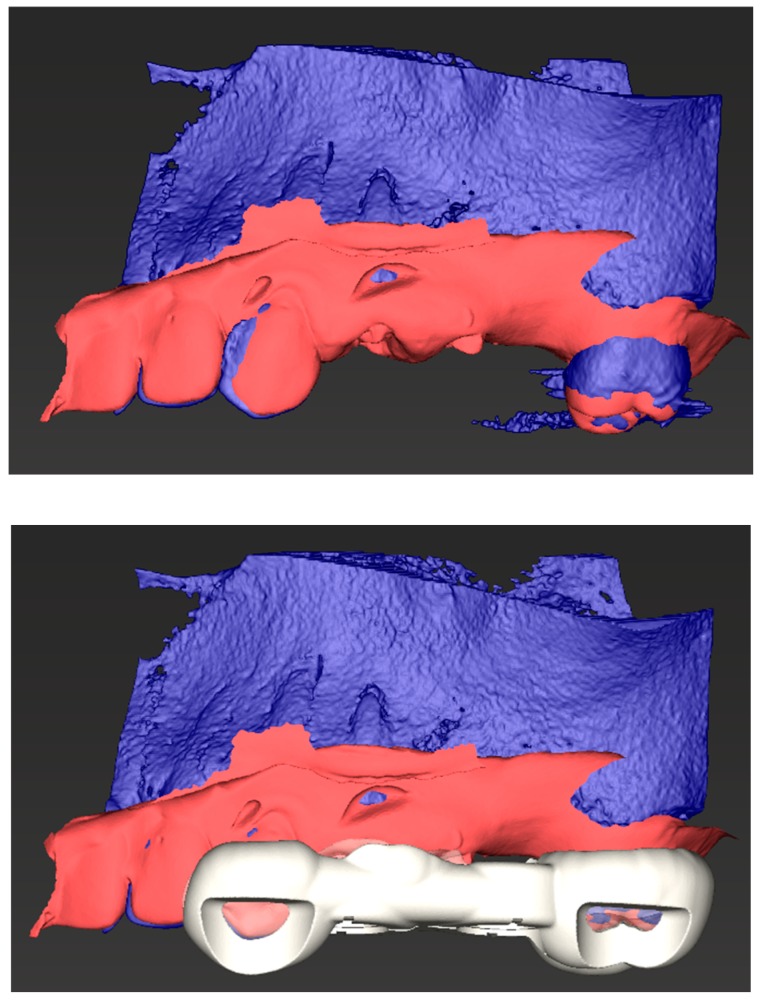
Combined stereolithography (STL) data with Digital Imaging and Communications in (DCM) data (**top image**) and STL exportable/printable surgical guide allowing for precision surgical entry (**bottom image**).

Improved image quality will advance elements of guided surgery, enabling clearer stereolithography (STL) and Digital Imaging and Communications in Medicine (DICOM or DCM) data merging with creation of surgical guides for minimally invasive surgery and calculated bone volumes for accurate guided tissue regeneration (GTR) bone volume requirements (Figure 4).

## 2. Conclusions

Despite the fact that there is rapidly accruing literature on CBCT, there are still no current evidence-based guidelines on its necessity and use for periodontal treatment planning. In selective cases, however, limited field of view CBCT may be useful for periodontal disease diagnoses due to less radiation dosage to the patient, higher spatial resolution, and shorter volumes to be interpreted. Further studies are justified to continue to evaluate the role of CBCT in the diagnosis and management of periodontal disease. Areas of interested include standardization of imaging protocols, regarding FOV and resolution requirements for accurate assessments. At this time, although evidence is emerging, it cannot be recommended as the standard of care and ultimately it is up to the individual clinician to use their own judgment as to when the additional information provided by CBCT may be beneficial, while always keeping the ALARA principle in mind.
